# Antiaging effect of dietary chitosan supplementation on glutathione-dependent antioxidant system in young and aged rats

**DOI:** 10.1007/s12192-012-0354-2

**Published:** 2012-07-25

**Authors:** R. Anandan, B. Ganesan, T. Obulesu, S. Mathew, K. K. Asha, P. T. Lakshmanan, A. A. Zynudheen

**Affiliations:** 1 Biochemistry and Nutrition Division, Central Institute of Fisheries Technology, Matsyapuri (PO), Cochin, 682029 Kerala India; 2 Fish Processing Division, Central Institute of Fisheries Technology, Matsyapuri (PO), Cochin, 682029 Kerala India

**Keywords:** Chitosan, Aging, Oxidative stress, Antioxidant status

## Abstract

Aging has been defined as the changes that occur in living organisms with the passage of time that lead to functional impairment and ultimately to death. Free radical-induced oxidative damage has long been thought to be the most important consequence of the aging process. In the present study, an attempt has been made to study the salubrious effects of dietary supplementation of chitosan on glutathione-dependent antioxidant defense system in young and aged rats. The dietary supplementation of chitosan significantly reduced the age-associated dyslipidemic abnormalities noted in the levels of total cholesterol, HDL-cholesterol, and LDL-cholesterol in plasma and heart tissue. Its administration significantly (*P* < 0.05) attenuated the oxidative stress in the heart tissue of aged rats through the counteraction of free radical formation by maintaining the enzymatic [glutathione peroxidase (GPx) and glutathione reductase (GR)] and non-enzymatic [reduced glutathione (GSH)] status at levels comparable to that of normal young rats. Our results conclude that dietary intake of chitosan restores the depleted myocardial antioxidant status and suggest that it could be an effective therapeutic agent in treatment of age-associated disorders where hypercholesterolemia and oxidative stress are the major causative factors.

## Introduction

Aging, a multifactorial process of enormous complexity, is characterized by impairment of physiochemical and biological aspects of cellular functions (Harman 1992). Oxidative stress, an unavoidable consequence in the metabolism of oxygen in aerobic cells, is a major factor in the aging process and, in the course of many chronic diseases, associated with aging (Mattson 2002). Many predisposing conditions which increase in prevalence during aging, such as obesity, insulin resistance, inflammation, changes in the activity of the hypothalamus–hypophysis suprarenal axis, stress, and hypertension, contribute to increase prevalence of cardiovascular diseases (Veronica and Esther 2012). Lipid infiltration in the myocardium is the foremost disorder encountered in the development of the aging process (Johannsen and Ravussin 2010). Aging is frequently accompanied by several pathological conditions and some associated phenomena such as increased lipid peroxidation, generation of free radicals, and increased peroxidation of nitric oxide (NO) to its toxic species, resulting from oxidative stress which significantly alters the incidence of cardiovascular diseases (Guarner et al. 2005). Alteration in glutathione-dependent antioxidant system is expected to exert a significant impact on physiological and metabolic functions of cellular membranes. Enhanced lipid peroxidation and deterioration of membrane structure has been well established during the aging process (Yu 2005). Preventing and treating cardiovascular diseases would be useful in promoting normal aging. The identification of natural molecule with antioxidant, antilipidemic, and membrane-stabilizing properties is, therefore, one strategy to facilitate healthy aging.

**Fig. 1:**
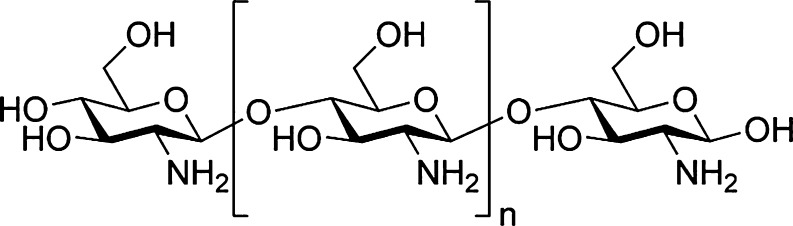
Structure of chitosan

Chitosan is one of the most abundant naturally occurring polysaccharides present in shellfish, clams, krill, oysters, squid, fungi, and insects (Cardenas et al. 2001). It is a polymer of α-(1-4)-d-glucosamine (Fig. 1), and it is chemically similar to that of the plant fiber, cellulose. It has been reported to possess antilipidemic (Santhosh et al. 2006), antioxidant (Xie et al. 2001), and membrane-stabilizing properties (Filipovic-Grcic et al. 2001). Previously, Anandan et al. (2004) observed the antiulcerogenic potential of chitosan against HCl–ethanol-induced peptic ulcer in rats. The free radical quenching property of this marine polysaccharide has also been studied in detail (Xing et al. 2005). Reports by Filipovic-Grcic et al. (2001) indicated the membrane-stabilizing property of chitosan. It has profound applications in the fields of clarification and purification, chromatography, paper and textiles, photography, food and nutrition, agriculture, pharmaceutical and medical, cosmetics, biodegradable membranes, and biotechnology (Santhosh et al. 2006). Though the beneficial effects of chitosan have been extensively studied, the antiaging effect of chitosan has not yet been explored.

In the present study, an attempt has been made to assess the salubrious effect of dietary chitosan intake on myocardial lipid peroxidation and glutathione-dependent antioxidant status in young and aged rats by virtue of its antioxidant and hypolipidemic properties.

## Experimental

### Drugs and chemicals

Reduced glutathione, tetraethoxypropane, and 2-thiobarbituric acid were procured from M/s Sigma Chemical Company, St. Louis, MO, USA. All other chemicals used were of analytical grade. Chitosan (Mw, 750,000 Da; viscosity, 8 cP; deacetylation rate, 85–87 %; purity, 98.6 %) used in the experiment was a kind gift from Dr. T. K. Thankkappan, Principal Scientist, Central Institute of Fisheries Technology, Cochin, India.

### Animals

Male Wistar strain albino rats, weighing 120–150 g [18 young rats of 2–3 months old (mean age, 79.2 ± 6.53 days)] and 350–400 g [18 aged rats of 20–25 months old (mean age, 712 ± 51.6 days)], were selected for the study. The animals were housed individually in polypropylene cages under hygienic and standard environmental conditions (28 ± 2 °C; humidity, 60–70 %; 12 h light/dark cycle). The animals were allowed a standard diet (M/s Sai Foods, Bangalore, India; Table 1) and water ad libitum. The experiment was carried out according to guidelines of the Committee for the Purpose of Control and Supervision of Experiments on Animals, New Delhi, India, and approved by the Institutional Animal Ethics Committee of the Central Institute of Fisheries Technology, Cochin, India.

**Table 1:** Composition of standard diet

S. No.	Ingredients	Composition (g/100 g diet)
1	Carbohydrate (nitrogen free)	56.2
2	Crude protein	22.0
3	Ash	7.5
4	Crude oil	4.2
5	Crude fiber	3.0
6	Glucose	2.5
7	Vitamins	1.8
8	Sand silica	1.4
9	Calcium	0.8
10	Phosphorus	0.6

### Experimental protocol

Seven days after acclimatization, the animals were divided into two major groups: Group I consisted of 18 normal young rats and Group II consisted of 18 normal aged rats. Each group was further subdivided into three groups (six rats each): one control group (Group Ia and Group IIa) and two experimental groups based on the duration of supplementation of chitosan at 2 % level along with feed 30 days (Group Ib and Group IIb) and 60 days (Group Ic and Group IIc).

At the end of the experimental period, the animals were sacrificed, and the blood was collected in a heparinized tube for the separation of plasma. The heart tissue was excised immediately and homogenized in ice-cold 0.1-M Tris–HCl buffer in a Potter–Elvehjem homogenizer. The homogenate was used for the estimation of lipid peroxides (LPO; Ohkawa et al. 1979), reduced glutathione (GSH; Ellman 1959), glutathione reductase (GR; Stall et al. 1969), and glutathione peroxidase (GPX; Paglia and Valentine 1967). High-density lipoprotein-cholesterol (HDL-cholesterol) in plasma was determined by the method of Izzo et al. (1981), and low-density lipoprotein-cholesterol (LDL-cholesterol) was estimated according to the calculation of Friedwald et al. (1972). The total cholesterol was estimated by the method of Allain et al. (1974) after extracting total lipids according to the method of Folch et al. (1957).

### Statistical analysis

Results are expressed as mean ± SD. Multiple comparisons of the significant ANOVA were performed by Duncan's multiple range comparison test. A *P* value <0.05 was considered as statistically significant. All data were analyzed with the aid of statistical package program SPSS 12.0 for Windows.

## Results and discussion

Significant (*P* < 0.05) variation was observed in the body weight (grams) of young (initial, 129 ± 9.05; final, 249 ± 18.3) and aged (initial, 382 ± 27.2; final, 336 ± 23.1) chitosan-supplemented groups of rats. The significant (*P* < 0.05) loss observed in the body weight of chitosan-supplemented aged animals might be related to the fibrous nature of chitosan. Interestingly, the total food intake (grams per 60 days) of aged chitosan-supplemented rats (904 ± 78.4) was comparable to that of young rats (745 ± 59.5). Level of total cholesterol was significantly (*P* < 0.05) higher in plasma and heart tissue of Group IIa aged rats as compared to Group Ia young control rats, indicating the development of mild age-associated hypercholesterolemic condition (Tables 2 and 3). The level of LDL-cholesterol was slightly (*P* < 0.05) higher in Group IIa aged rats, whereas HDL-cholesterol levels were significantly lower compared to Group Ia young animals (Table 2). This aspect might be due to the augmented mobilization of LDL-cholesterol from the blood into the cell membranes, resulting in abnormal cholesterol deposition in the myocardium. In the present study, the dietary supplementation with chitosan significantly reduced the total cholesterol level in plasma and myocardial tissue of Group IIc aged rats as compared to Group IIa rats. It also kept the levels of LDL-cholesterol and HDL-cholesterol in plasma comparable to that of Group Ic rats. The present observations concurs with an earlier reported study (Baker et al. 2009), which showed that the positively charged amino groups of chitosan possess the ability to bind negatively charged molecules such as lipids and bile acids, inducing a greater fractional excretion in the feces. Also, studies by Xu et al. (2007) suggested that chitosan improve lipid metabolism by regulating total cholesterol and LDL-cholesterol by upregulation of hepatic LDL receptor mRNA expression, increasing the excretion of fecal bile acids. Previous studies (Yao and Chiang 2002) pointed out that chitosan supplementation was capable of lowering the levels of plasma total cholesterol and LDL-cholesterol in experimental animals. In the present study, a slight decline in the level of total cholesterol and LDL-cholesterol were also noted in Group Ic chitosan-fed young rats, ascertaining the anticholesterolemic property of chitosan (Ylitalo et al. 2002).

**Table 2:** Effect of dietary chitosan supplementation on total cholesterol, HDL-cholesterol, and LDL-cholesterol in plasma of young and aged rats

Parameters	Young rats			Aged rats		
	Group Ia (control)	Group Ib (30 days)	Group Ic (60 days)	Group IIa (control)	Group IIb (30 days)	Group IIc (60 days)
Total cholesterol	79.9 ± 5.36 a,b	76.2 ± 4.98 a,b	73.1 ± 5.07 a	98.4 ± 9.12 c	91.7 ± 8.56 c	87.3 ± 8.21 b,c
HDL-cholesterol	42.2 ± 3.11 a,b,c	44.9 ± 3.48 b,c	47.5 ± 3.29 c	33.2 ± 2.68 d	36.4 ± 2.56 a	39.7 ± 3.74 a,b
LDL-cholesterol	24.3 ± 1.42 a	22.7 ± 1.31 a,b	20.3 ± 1.18 b	48.4 ± 2.72 c	43.1 ± 2.14 d	38.3 ± 2.27 e

Results are mean ± SD for six rats. Values expressed: total cholesterol, HDL-cholesterol, and LDL-cholesterol, milligrams per deciliter. Values that have a different letter (a, b, c, d, e, f) differ significantly with each other (*P* < 0.05; Duncan's multiple range test)

**Table 3:** Effect of dietary chitosan supplementation on the levels of total cholesterol, lipid peroxides, and reduced glutathione (GSH) and the activities of glutathione-dependent antioxidant enzymes [glutathione peroxidase (GPx) and glutathione reductase (GR)] in the heart tissue of young and aged rats

Parameters	Young rats			Aged rats		
	Group Ia (control)	Group Ib (30 days)	Group Ic (60 days)	Group IIa (control)	Group IIb (30 days)	Group IIc (60 days)
Total cholesterol	2.72 ± 0.17 a,b	2.56 ± 0.14 a	2.43 ± 0.16 a	3.68 ± 0.32 c	3.29 ± 0.21 d	3.04 ± 0.25 b,d
Lipid peroxides	1.08 ± 0.07 a	0.98 ± 0.06 a	0.97 ± 0.07 a	2.58 ± 0.14 b	1.96 ± 0.09 c	1.54 ± 0.11 d
GSH	10.9 ± 0.84 a	12.3 ± 1.02 a,b	14.5 ± 1.17 c	7.54 ± 0.61 d	8.76 ± 0.69 d,e	9.52 ± 0.85 a,e
GPx	5.23 ± 0.35 a	5.51 ± 0.41 a,b	5.98 ± 0.37 b	3.12 ± 0.18 c	4.05 ± 0.26 d	4.56 ± 0.34 d
GR	0.43 ± 0.02 a	0.52 ± 0.04 b	0.58 ± 0.03 c	0.22 ± 0.01 d	0.29 ± 0.03 e	0.36 ± 0.02 f

Results are mean ± SD for six animals. Values expressed: total cholesterol, milligrams per gram wet tissue; lipid peroxides, nanomoles MDA released per milligram protein; GSH, micrograms per milligram protein; GPx, micrograms GSH oxidized per minute per milligram protein; GR, nanomoles NADPH oxidized per minute per milligram protein. Values that have a different letter (a, b, c, d, e, f) differ significantly with each other (*P* < 0.05; Duncan's multiple range test)

Sumiyoshi and Kimura (2006) suggested that the lipid-lowering effects of chitosan might be mediated by increases in fecal fat and/or bile acid excretion resulting from the binding of bile acids, and by a decrease in the absorption of dietary cholesterol from the small intestine. Studies by Maslowski et al. (2009) have shown that normal intestinal microbiota might also positively influence immune responses and protect against the development of inflammatory diseases systemically through the formation of short-chain fatty acids by fermentation of dietary fiber in the intestine. Chitosan, a biopolymer of glucosamine derived from chitin that is chemically similar to that of cellulose, acts as a dietary fiber in gastrointestinal tract (Gallaher et al. 2000). It is possible that chitosan may function through the generation of gut metabolites, such as short-chain fatty acids/short-chain oligosaccharides, in attenuating the development of inflammatory processes related to aging.

In the present study, there was a significant (*P* < 0.05) increase in the level of lipid peroxidation observed with a concomitant reduction in the level of nonenzymatic (GSH) and enzymatic (GPx and GR) antioxidants in the heart tissue of Group IIa aged rats as compared to Group Ia young control animals (Table 3). This concurs with the earlier findings (Subramanian and James 2010), which indicated that the higher vulnerability of aged myocardium to peroxidative damage was mainly due to a decline in the level of free radical scavengers. Depletion of GSH results in enhanced lipid peroxidation, and excessive lipid peroxidation can cause increased GSH consumption during aging (Denniss et al. 2011), as observed in the present study. GSH protects the cardiac cell membranes from the damaging action of lipid peroxide. The reduction in the activity of GPx and GR may be due to the reduced availability of GSH. GPx offers protection to the cellular and subcellular membranes from the peroxidative damage by eliminating hydrogen peroxide and lipid peroxide (Li et al. 2005). Inhibition of GSH-dependent antioxidant enzymes makes myocardial cell membranes more susceptible to oxidative damage in aging. GSH and GSH-dependent enzyme systems may be directly related to the pathogenic mechanisms related to age-associated disorders (Goncharova et al. 2007).

In our study, the dietary chitosan intake significantly attenuated the age-associated oxidative stress and maintained the level of the glutathione-dependent antioxidant status in the heart tissue at near normal. It probably did so by its antioxidant nature. Reports by Jeon et al. (2003) have shown that chitosan has strong antioxidative effects, which decrease free radical production and increase antioxidant enzyme activities during CCl_4_-induced lipid peroxidation in rats. Je et al. (2004) have suggested that chitosan may eliminate various free radicals by the action of nitrogen on the C-2 position of the chitosan. Xie et al. (2001) reported that the scavenging mechanism of chitosan is related to the fact that the free radicals can react with the hydrogen ion from the ammonium ions to form a stable molecule. The normal young rats receiving chitosan (Group Ic) did not show any significant change when compared with normal (Group Ia) rats, indicating that it does not per se have any adverse effects.

In conclusion, the overall antiaging effect of dietary chitosan intake is probably related to its ability to inhibit the increased accumulation of lipids both in the systemic circulation and in the myocardium by its antilipidemic property, or to normal maintenance of the activities of glutathione-dependent antioxidant enzymes and the level of GSH, which protect myocardial membrane against oxidative stress by decreasing lipid peroxidation reactions.
